# Development and validation of a nomogram to predict coronary heart disease in patients with rheumatoid arthritis in northern China

**DOI:** 10.18632/aging.102823

**Published:** 2020-02-29

**Authors:** Tingting Wei, Bowen Yang, Haina Liu, Fangran Xin, Lingyu Fu

**Affiliations:** 1 Department of Clinical Epidemiology and Evidence-based Medicine, The First Affiliated Hospital, China Medical University, Shenyang, China; 2 Department of Medical Record Management Center, The First Affiliated Hospital, China Medical University, Shenyang, China; 3 Department of Rheumatology, The First Affiliated Hospital, China Medical University, Shenyang, China

**Keywords:** rheumatoid arthritis, coronary heart disease, Framingham risk score, development and validation nomogram

## Abstract

We developed and validated a nomogram to predict coronary heart disease (CHD) in patients with rheumatoid arthritis (RA) in northern China. We analyzed a cohort of RA patients admitted to the Department of Rheumatology and Immunology of the First Affiliated Hospital of China Medical University from 2011 to 2017. To select a high-performance model for clinical data prediction, we evaluated the F1-scores of six machine learning models. Based on the results, we selected multivariable logistic regression analysis for the development of a prediction model. We then generated an individualized prediction nomogram that included age, sex, hypertension, anti-cyclic citrullinated peptide antibody positivity, the erythrocyte sedimentation rate, and serum LDL-cholesterol, triglyceride and HDL-cholesterol levels. The prediction model exhibited better discrimination than the Framingham Risk Score in predicting CHD in RA patients. The area under the curve of the prediction model was 0.77, with a sensitivity of 63.9% and a specificity of 77.2%. The nomogram exhibited good calibration and clinical usefulness. In conclusion, our prediction model was more accurate than the Framingham Risk Score in predicting CHD in RA patients. Our nomogram combining various risk factors can be used for the individualized preoperative prediction of CHD in patients with RA.

## INTRODUCTION

Rheumatoid arthritis (RA) is the prototype of chronic inflammatory rheumatic disease, and is associated with accelerated atherosclerosis and an increased risk of cardiovascular disease (CVD) [1–3]. Patients with RA are at greater risk for coronary heart disease (CHD) than those without RA [4]. Cardiovascular events occur in 40-50% of patients with RA, and have become the leading cause of death in RA patients [5, 6]. Thus, it is important to be able to predict whether patients with RA are at high risk for cardiovascular events so that preventive measures can be taken. While some traditional CHD risk factors (e.g., hypertension [7], disease activity [8], smoking [9] and dyslipidemia [10]) are more prevalent among RA patients than among those without RA, these factors explain only a portion of the observed excess of CHD risk in RA patients. In fact, the incidence of atherosclerosis is two to three times higher in RA patients than in the general population after adjustment for traditional risk factors of atherosclerosis [11–13]. Therefore, the predictors of CHD in RA have not been fully elucidated.

Inflammation and immunization are intrinsic components of RA, and are key accelerators of cardiovascular risk in RA patients [11, 14]. The anti-cyclic citrullinated peptide (anti-CCP) antibody may also be associated with the pathogenesis, clinical expression and cardiovascular risk of RA patients [15–17]. Despite the association of inflammation, immunization and other biological indicators with increased CVD risk in RA patients, these factors have not been included in many CVD risk assessment tools. The Framingham risk score (FRS) is the most widely used tool for predicting the risk of incident CVD events over 10 years in the general population [18]. However, since the FRS contains only traditional risk factors, it may underestimate the risk of CVD in RA patients [19]. Thus, it is necessary to explore other bio-predictive markers and to further refine the CHD risk prediction models for RA patients.

Combined analyses of various biomarkers, rather than separate analyses, are sufficient to determine appropriate, individualized prevention methods for patients. Therefore, in this study, we sought to develop and validate a nomogram that incorporated serum lipid levels, inflammatory marker levels and serological status data for the individualized prediction of CHD in RA patients.

## RESULTS

### Clinical characteristics

In this study, we analyzed a cohort of RA patients with and without CHD who were admitted to the Department of Rheumatology and Immunology of the First Affiliated Hospital of China Medical University from 2011 to 2017. We divided the total cohort of patients into a training cohort (n=1012) and a validation cohort (n=274). We then assessed the clinical characteristics and serological statuses of patients in the training and validation cohorts, and performed univariate analysis to examine the differences between RA patients and RA+CHD patients in each cohort, including a chi-square tests and t tests (Table 1). There were no significant differences in mean age and sex between RA patients and RA+CHD patients in either the training cohort or the validation cohort. In the training cohort, the age distribution, hypertension, anti-CCP antibody positivity, rheumatoid factor positivity, erythrocyte sedimentation rate (ESR), C-reactive protein (CRP) levels, and dyslipidemia of low-density lipoprotein cholesterol (LDL-c), total cholesterol (TC), triglycerides and high-density lipoprotein cholesterol (HDL-c) differed significantly between the RA and RA+CHD groups (all *P*<0.10). In the validation cohort, the age distribution, hypertension, anti-CCP antibody positivity, rheumatoid factor positivity, ESR, and dyslipidemia of LDL-c, TC, triglycerides and HDL-c differed significantly between the RA and RA+CHD groups (all *P*<0.10), while CRP levels did not differ between the two groups in this cohort.

**Table 1:** Characteristics of Patients in the training group and the validation group.

	**Training group**			**Validation group**		
	**RA+CHD(294)**	**RA(718)**	***P***	**RA+CHD(70)**	**RA(204)**	***P***
**Demographic data**						
Age(year)	69.7(39,92)	61.58(20,93)	0.000	68.84(36,90)	62.24(23,90)	0.001
Sex (male, %)	65(22.1)	159(22.1)	1.000	17(28.8)	42(20.6)	0.505
Smoking, n (%)	49(16.7)	103(14.3)	0.384	9(12.9)	36(17.6)	0.455
Hypertension(%)	142(48.3)	163(22.7)	0.000	39(55.7)	41(20.1)	0.000
**Dyslipidemia**						
LDL(mmol/L)	3.05(0.61,7.32)	2.59(0.52,6.39)	0.000	2.86(1.16,4.44)	2.63(0.31,10.23)	0.057
TC(mmol/L)	4.78(0.72,9.41)	4.19(1.72,8.62)	0.000	4.68(2.75,9.2)	4.26(1.72,11.8)	0.008
HDL(mmol/L)	1.16(0.14,3.07)	1.07(0.14,2.64)	0.001	1.18(0.62,2.22)	1.06(0.11,2.64)	0.030
TG(mmol/L)	1.48(0.31,7.22)	1.22(0.25,11.23)	0.000	1.52(0.44,5.94)	1.3(0.33,6.19)	0.076
**Serologic profile**						
Anti-CCP-positive(%)	184(62.6)	353(49.2)	0.000	46(65.7)	108 (52.9)	0.070
RF-positive(%)	220(74.8)	454(63.2)	0.000	55(78.6)	135(66.2)	0.071
ESR(mm/h)	49.65(2,138)	43.59(2,132)	0.001	58.14(7,145)	47.52(2,128)	0.004
CRP(mg/L)	37.21(0.11,266)	41.84(0.14,310)	0.020	41.65(0.21,249)	43.39(0.23,320)	0.684
C4(g/L)	0.22(0.02,0.5)	0.22(0.02,1.13)	0.918	0.22(0.06,0.39)	0.22(0.02,0.55)	0.937
C3(g/L)	1.11(0.28,1.94)	1.09(0.19,2.39)	0.336	1.08(0.54,1.71)	1.06(0.25,1.96)	0.545
**Treatment**			0.341			0.941
Biological drugs(%)	40(13.3)	76(10.2)		9(14.5)	29(16.4)	
Non-biological drugs(%)	249(83.0)	639(85.8)		47(75.8)	131(74.0)	
Other drugs(%)	11(3.7)	30(4.0)		6(9.7)	17(9.6)	

Abbreviation: LDL, low-density lipoprotein cholesterol; TC, total cholesterol; TG, triglycerides; HDL, high-density lipoprotein cholesterol; RF-positive, positive rheumatoid factor; CRP, C-reactive protein; Anti-CCP-positive, positive anti-cyclic citrullinated peptide antibody; ESR, erythrocyte sedimentation rate; C3, complement 3; C4, complement 4.

### Model evaluation

**Figure 1:**
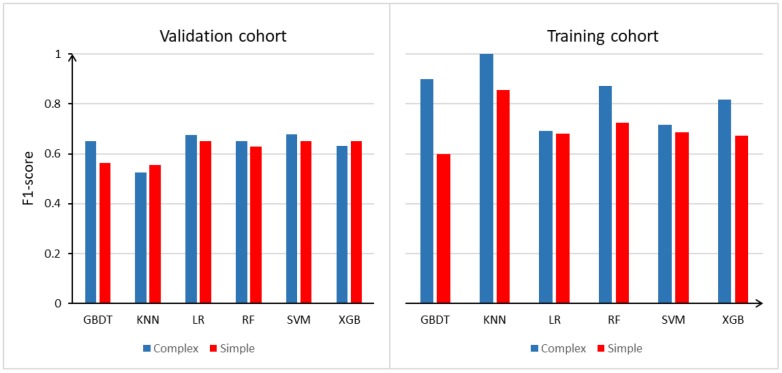
**Model evaluation (F1-score) results based on the number of features across 6 models in training group and validation group.** Abbreviation: GBDT: gradient boosting decision tree; KNN: k-nearest-neighbors; LR: logistic regression; RF: random forest; XGB: xgradient-boosting; SVM: support vector machine.

In order to select a high-performance model for clinical data prediction, we evaluated the F1-scores of six machine learning models: gradient boosting decision tree (GBDT), k-nearest-neighbors (KNN), logistic regression (LR), random forest (RF), XGradient-boosting (XGB) and support vector machine (SVM). Figure 1 displays the F1-score for each model in the training and validation cohorts. The LR model performed at the same level as the other machine learning models, based on its F1-score and area under the curve (AUC), indicating that the LR algorithm was an effective prediction tool for the current data (Supplementary Table 1). In the training cohort, the KNN algorithm displayed better prediction performance than the other algorithms, which may have been due to the algorithm itself, and there was an over-fitting phenomenon. However, in the validation group, the KNN algorithm exhibited poor prediction effects, indicating that this algorithm was unstable. The SVM and LR algorithms had more stable performance, and the LR algorithm could be used to construct a nomogram. Therefore, the LR algorithm was used for clinical data prediction.

In addition, we constructed a simple model based on the training and validation cohorts. We also constructed a complex model of the RA and RA+CHD patients in the training and validation cohorts by adjusting the simple model for age and sex. Based on the F1-scores and AUCs in the training and validation cohorts, the average performance of the complex model was better than that of the simple model, indicating that sex and age are associated with the risk of CHD in patients with RA. Thus, the inclusion of these factors could improve the accuracy of CHD risk prediction.

### Risk factors for the development of CHD in RA patients

To further explore the independent risk factors for CHD in RA patients, we carried out a non-conditional LR analysis based on the results of our univariate analysis (Supplementary Table 2). First, we constructed a simple model of RA patients and RA+CHD patients in the training cohort. The independent risk factors for the development of CHD among RA patients in the training cohort were hypertension (OR=3.06, 95% CI: 2.26-4.15, *P*<0.001), rheumatoid factor positivity (OR=1.48, 95% CI: 1.04-2.11, *P*=0.031), high LDL-c levels (OR=1.85, 95% CI: 1.25-2.72, *P*=0.002), low HDL-c levels (OR=2.17, 95% CI: 1.26-3.76, *P*=0.006), high triglyceride levels (OR=1.41, 95% CI: 1.14-1.74, *P*=0.002) and a high ESR (OR=1.01, 95% CI: 1.00-1.01, *P*=0.001).

We also constructed a complex model of RA patients and RA+CHD patients in the training cohort by adjusting the simple model for age and sex. We found that age (OR=1.05, 95% CI: 1.04-1.07, *P*<0.001), hypertension (OR=2.95, 95% CI: 2.15-4.05, *P*<0.001), anti-CCP antibody positivity (OR=1.41, 95% CI: 1.00-1.97, *P*=0.047), high LDL-c levels (OR=1.95, 95% CI: 1.31-2.91, *P*=0.001), low HDL-c levels (OR=2.04, 95% CI: 1.16-3.61, *P*=0.013), high triglyceride levels (OR=1.47, 95% CI: 1.19-1.81, *P*<0.001) and a high ESR (OR=1.01, 95% CI: 1.00-1.01, *P*=0.009) were independent risk factors for the development of CHD among RA patients in the training cohort.

### Development of an individualized prediction model

**Figure 2:**
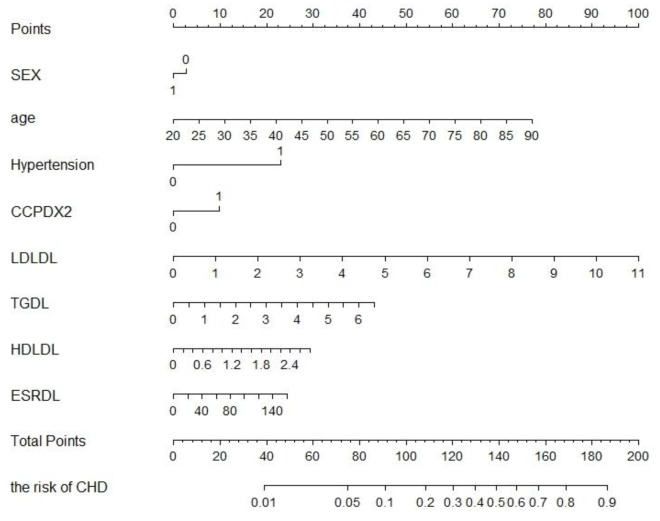
**RA patients developed to CHD’s nomogram.** The CHD nomogram was developed in the training cohort, with serum lipids, inflammatory markers, and serological status in RA patients. Abbreviation: LDL, low-density lipoprotein cholesterol; TC, total cholesterol; TG, triglycerides; HDL, high-density lipoprotein cholesterol; RF+, positive rheumatoid factor; CRP, C-reactive protein; Anti-CCP-positive, positive anti-cyclic citrullinated peptide antibody; ESR, erythrocyte sedimentation rate.

Our univariate analysis identified age, hypertension, anti-CCP antibody positivity, rheumatoid factor positivity, a high ESR, high CRP levels, and dyslipidemia of LDL-c, TC, triglycerides and HDL-c as independent predictors of CHD (Table 1). It is worth noting that in univariate analysis, the relationships between certain independent and dependent variables may be masked by confounding factors, so variables with *P* values <0.100 were entered into a multi-factor model analysis. Complicated models combined with the above independent predictors were developed into nomograms Complicated models combined with the above independent predictors were developed into nomograms (Figure 2). The figure displays the score associated with the level of each influencing factor, the personal total score (the sum of the scores for all the factors) and the predicted risk of CHD for the RA patient. For example, an RA patient who was 65 years old (48.5 points) and male (2.5 points), with hypertension (23.0 points), 2.0 mmol/L LDL-c (17.5 points), 2.5 mmol/L triglycerides (16.5 points), 2.4 mmol/L HDL-c (25.0 points), 80 mm/h ESR (12.5 points) and anti-CCP antibody positivity (10.0 points) would have a total points value of 144.5, resulting in an estimated probability of 58% for CHD.

### Validation of the nomogram

**Figure 3:**
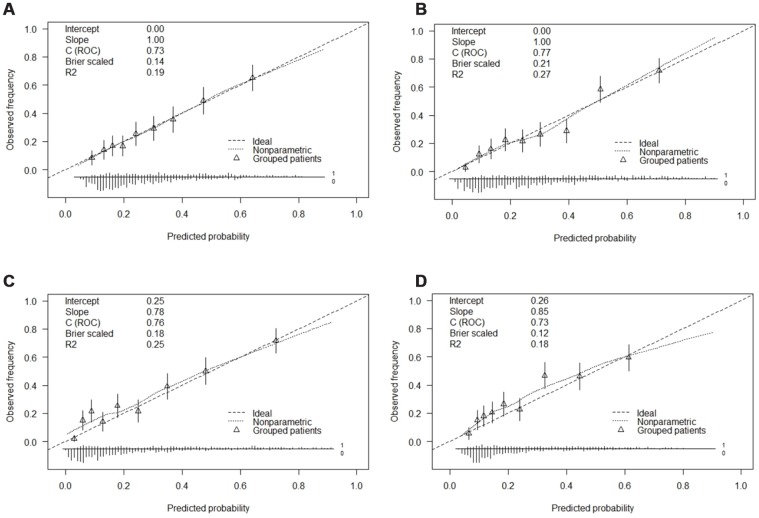
**Calibration curves of the CHD and the model with the addition of sex and age prediction in each cohort.** Abbreviation: (**A**) Calibration curve of the CHD in the simple model of the development cohort. (**B**) Calibration curve of the complex model with addition of adjusted sex and age in the development cohort. (**C**) Calibration curve of the CHD in the simple model of the validation cohort. (**D**) Calibration curve of the complex model with addition of adjusted sex and age in the validation cohort.

The calibration curves of the nomogram for CHD development in RA patients indicated that there was good agreement between the predicted and observed outcomes in the training cohort (Figure 3A and 3B). The concordance index (C-index) of the prediction nomogram was 0.73 when the simple model was used and 0.77 when the complex model was used. Similarly, there was good agreement between the predicted and observed outcomes in the validation cohort (Figure 3C and 3D). The C-index of the nomogram for the prediction of CHD was 0.76 with the simple model and 0.73 with the complex model.

### Sensitivity and specificity of the FRS and our prediction models

**Figure 4:**
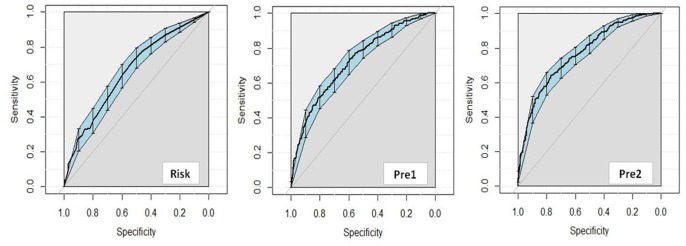
**Diagnostic value for FRS and the clinical prediction model to predict CHD.** Abbreviation: FRS, diagnostic value for FRS to predict CHD; Pre1, diagnostic value for simple clinical prediction model to predict CHD; Pre2, diagnostic value for complex clinical prediction model to predict CHD.

We next constructed receiver operating characteristic curves for the simple model, the complex model and the FRS (Figure 4). Based on the AUCs, the complex prediction model (AUC: 0.77, 95% CI: 0.74-0.80, *P*<0.000) had a better diagnostic value than the simple prediction model (AUC: 0.73, 95% CI: 0.69-0.76, *P*<0.000) and the FRS (AUC: 0.66, 95% CI: 0.62-0.70, *P*<0.000) in predicting the development of CHD in RA patients. The sensitivity of the simple prediction model (73.8%) was higher than those of the complex prediction model (63.9%) and the FRS (71.8%). However, the specificity of the complex prediction model (77.2%) was higher than those of the simple prediction model (59.2%) and the FRS (52.4%) (Supplementary Table 3).

### Clinical use

**Figure 5:**
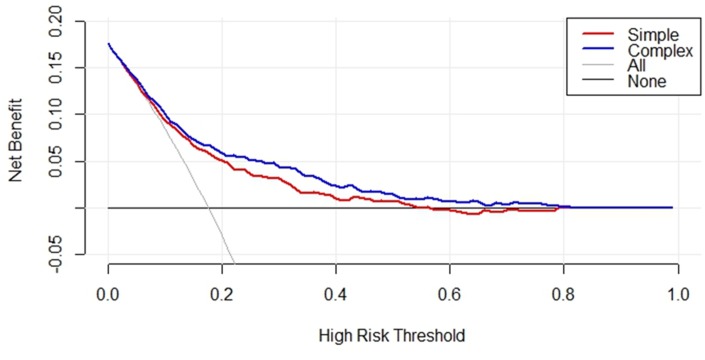
**Decision curve analysis for serum lipids, inflammatory markers, and serological status in RA and CHD patients of the simple and complex model in the training cohorts.** The y-axis represents the net benefit, the x-axis represents the high-risk threshold of CHD in RA patients. The red line represents the nomogram of predictors in simple model. The blue line represents the complex model with addition of sex and age. The gray line represents the assumption that all patients have CHD.

Next, we performed a decision curve analysis for the nomogram, as presented in Figure 5. The decision curve demonstrated that if the threshold probability of a patient or doctor was <19%, using the nomogram to predict CHD was more beneficial than either the treat-all-patients scheme or the treat-none scheme. Within this range, the net benefits of the simple and complex models were comparable, with several overlaps. However, when the threshold value was >19%, the net benefit of the complex model (blue line) was higher than that of the simple model (red line) in predicting the risk of CHD in RA patients.

### Clinical impact curve

A clinical impact curve can be used to demonstrate the clinical effectiveness of a model by predicting the risk stratification of 1000 people with the bootstrap technique. We drew clinical impact curves for the simple and complex models to determine their cost-benefit ratios, as shown in Supplementary Figure 2. The figure displays the number of high-risk patients (the number of positive cases predicted by the model) and the number of high-risk patients with events (the number of true-positive cases). In this example, the dotted vertical line illustrates a tentative cut-off point (18% risk of CHD) at which 400 of 1000 men (40%) would be filtered by the simple model, with about 160 of these (40%) being true CHD cases. At this same cut-off point, 380 of 1000 men (38%) would be filtered by the complex model, with about 160 of these (42%) being true CHD cases.

## DISCUSSION

To improve the identification of RA patients at risk for developing CHD, we developed and validated a risk assessment model that uses clinical features and serological indicators to estimate CHD risk accurately and comprehensively. This is the first prediction model to assess the development of CHD in RA patients from northern China using electronic medical record (EMR) data from the real world. It is worth noting that the National Cholesterol Education Program Adult Treatment Panel III guidelines suggest that diabetes is a risk equivalent of CHD. Therefore, we excluded RA patients with diabetes. To ensure the validity of the model established in this study, we evaluated six machine learning models. The LR algorithm performed well in the evaluation and exhibited better generalization than the other models.

CVD is the main cause of death in RA patients, but the CVD risk prediction scores used in the general population cannot accurately predict the risk of CVD in RA patients. Therefore, many researchers have developed and validated CVD risk prediction scores specifically for RA patients. An adjusted and recalibrated version of the European Systematic Coronary Risk Evaluation algorithm [20] (SCORE) was developed for RA patients in the Netherlands [21]. The new score included inflammatory disease activity and recalibrated traditional risk factors, but performed only slightly better than the original version in RA patients. Solomon et al. established an expanded cardiovascular risk prediction score for RA patients (ERS-RA), and suggested that traditional CVD risk factors could be used to predict CVD events in their cohort. However, the ERS-RA also included RA-related indicators such as inflammatory disease activity, disease course, daily prednisone dosage and so on [22]. Nevertheless, when the ERS-RA was externally validated, it did not perform better than FRS.

RA is a complex multi-system disease. Recent studies have demonstrated that multiple biochemical indexes can be analyzed in a single combined index. In this study, we used real-world data, combined with traditional atherosclerotic risk factors and RA-related indicators, to construct a more suitable predictive model of CHD risk in RA patients in northern China. Our prediction model combined multiple indicators (including age and sex), and exhibited a sufficient discriminatory capacity in both the training cohort (C-index, 0.77) and the validation cohort (C-index, 0.73).

We then established a nomogram that included traditional risk factors such as dyslipidemia and hypertension to predict the risk of CHD among RA patients. These traditional risk factors have been confirmed in previous studies on CVD among RA patients [23, 24]. In addition to traditional risk factors, inflammatory factors and anti-CCP antibody positivity were also found to be independent risk factors for CHD in RA patients in our study. Our findings underscored the important contribution of systemic inflammation to the development of CHD in RA patients by demonstrating that the ESR was independently associated with cardiovascular outcomes and mortality [14]. Moreover, our study suggested that serum anti-CCP antibody positivity may be an independent risk factor for the development of CHD in RA patients. Similar results have been reported in Mexico [25] and the US [26]. In addition, Lopez Longo et al. [27] found that anti-CCP antibody positivity was associated with endothelial dysfunction in RA patients.

Risk models for RA patients may need to include factors that are different from or additional to those for the general population, especially immune markers. Since the FRS does not include immune-related CVD risk factors such as the ESR and CRP levels, it may underestimate the CVD risk of RA patients. We compared the diagnostic value of our prediction model with that of the FRS, and found that our model had a higher AUC in predicting CHD in RA patients. The FRS substantially underestimated the CHD risk in RA patients with anti-CCP antibody positivity, rheumatoid factor positivity and persistently elevated ESRs. This indicates that RA disease severity and inflammation influence CHD risk in a manner that is not accounted for in the FRS. The poor discrimination and calibration of the FRS among RA patients could result in missed opportunities for preventive interventions and provide a false sense of security regarding CVD risk for both patients and their physicians.

Our study had several limitations. First, this study was performed at a single center in a hospital. The size of our study population was limited, and the results should be confirmed in a randomized controlled trial. Additionally, patients with CHD risk equivalents such as diabetes mellitus and peripheral vascular diseases were excluded from this study. Third, this study had the inevitable limitations of a retrospective study. There may have been a lag in the diagnosis of CHD in the RA + CHD patients, even if the CHD was present before the onset of RA. A prospective study would be required to avoid the interference of these factors. The risk of CHD in patients with diabetes mellitus requires further study. Finally, a follow-up study will be necessary to confirm the predictive power of the FRS for CHD in patients with RA.

In conclusion, we have presented a nomogram that incorporates serum lipid levels, inflammatory marker levels and the serological status, and can be conveniently used for the individualized prediction of CHD in patients with RA. Our prediction model was more accurate than the FRS in predicting CHD in RA patients. In the future, more detailed studies with larger sample sizes and external verifications should be designed to further improve and confirm the accuracy of our model.

## MATERIALS AND METHODS

### Subjects

This retrospective study was based on the EMRs of patients admitted to the inpatient Department of Rheumatology and Immunology of the First Affiliated Hospital of China Medical University between January 2011 and December 2017. The EMRs were classified and coded in accordance with the 10^th^ edition of the International Classification of Diseases. The code for RA is M05.x-06.x, and the code for CHD is I25.x.

All patients fulfilled the American College of Rheumatology 1987/2010 criteria for RA and/or the American Heart Association guidelines for CHD. RA+CHD patients met the following inclusion criteria: (1) conformity to the above RA diagnostic criteria; (2) conformity to the above CHD diagnostic criteria; and (3) over 18 years old. The onset times of RA and CHD were judged based on the durations of RA and CHD recorded in the EMRs, as well as the epidemiological investigation of the patient’s disease history by telephone follow-up and professional clinicians’ assessment. If the duration of CHD was shorter than the duration of RA, we inferred that the patient had developed CHD after being diagnosed with RA, and included the patient in the RA+CHD group. RA patients without CHD met the following inclusion criteria: (1) conformity to the above RA diagnostic criteria; (2) over 18 years old. Patients were excluded if they met the following criteria: (1) presence of diabetes; (2) presence of connective tissue diseases, including systemic lupus erythematosus, scleroderma and dry syndrome; and (3) lack of laboratory assessment data such as the levels of lipid markers, inflammatory markers or antibodies associated with RA.

Based on the inclusion and exclusion criteria, 364 RA+CHD patients and 922 RA patients were included in our study. The screening flow chart is shown in Supplementary Figure 1. We divided the study cohort into a training cohort and a validation cohort, and used 10-fold cross-validation to obtain a reliable and stable model. The training cohort, a random sample of 80% of the total cohort, was used to construct the prediction model of CHD in RA patients. The remaining 20% of the total cohort was designated as the internal validation cohort and used to determine the calibration and discrimination of the risk score. Ultimately, there were 1012 patients in the training cohort (294 RA+CHD patients and 718 RA patients) and 274 patients in the validation cohort (70 RA+CHD patients and 204 RA patients).

### Data collection

From the original EMRs, we collected data on demographics and health behaviors, including age, sex, disease course, menstruation (for women), body mass index, smoking habits and comorbidities. Subjects were categorized as having smoking habits (that is, long-term regular smoking of one cigarette per day) or hardly smoking (that is, never smoking or having quit smoking for one year). It is worth noting that missing data such as height, weight and menopausal history were obtained by telephone follow-up. The results of all clinical analyses of anti-CCP antibody positivity, rheumatoid factor positivity, complement component 3 levels, complement component 4 levels, the ESR, CRP levels and serum lipid measures (LDL-c, HDL-c, TC and triglyceride levels) were also obtained from the EMRs.

### Framingham risk score

The FRS, which incorporates age, gender, smoking status, blood pressure, TC and HDL-c levels, was used for CHD risk assessment. We excluded the presence of CHD risk equivalents (diabetes) identified in the National Cholesterol Education Program Adult Treatment Panel III guidelines [28].

### Simple model and complex model

Women are more likely to develop RA than men, possibly due to the higher estrogen levels in women. Therefore, sex is an important variable in the study of risk factors for RA. In addition, CHD is an age-related disease, so the variable of age could not be ignored in this study. Therefore, two models were constructed: a simple model that did not include the variables of sex and age, and a complex model that did. By comparing the two models, we could assess the contribution of age and gender to CHD in patients with RA.

### Statistical analysis

After assembling the study cohort, we performed a univariate analysis to compare the baseline characteristics between the RA and RA+CHD groups in both the training cohort and the validation cohort. Normality tests and descriptive statistical analyses (mean, percentage, maximum, minimum, etc.) were performed on the serological indicators, blood lipids, inflammatory markers and RA antibodies, and the differences between the two groups were analyzed by the chi-square test, t test or non-parametric test.

Next, a machine learning experiment was performed based on the scikit-learn open source algorithm library. The machine learning evaluation models adopt Neusoft's automatic medical analysis platform, and the parameters are selected according to the best parameters automatically given by the platform. A Bayesian Optimization method was used for algorithm tuning, and cross-validation was used to evaluate the algorithm. In the evaluation, we set the N-folds value to 5, selected LR, SVM, RF, XGB, GBDT and KNN for algorithm comparison, used a single-experiment design for each algorithm for 30 minutes, and performed a total of three experiments. Data from the training cohort and the validation cohort were evaluated in each experiment. The evaluation indicators included the AUC, F1-score, accuracy, precision, recall rate and balance error (ber), and the final result of the experiment was three secondary experimental averages.

Based on the results of the univariate analysis and machine learning experiment, LR analysis was used to analyze the risk factors of CHD in RA patients in the simple and complex models of the training cohort. We selected prediction indicators to build the prediction models based on our univariate analysis. To provide clinicians with a quantitative tool to predict an individual RA patient’s probability of developing CHD, we built a nomogram based on the LR analysis of the training cohort. The potential associations of the prediction indicators were first assessed in the training cohort and then validated in the validation cohort. A calibration curve was drawn to evaluate the calibration of the nomogram, and the Hosmer Lemeshow test was performed. The recognition performance of the nomogram was quantified based on the C-index, which was measured by the bootstrap method with 1000 resamplings. A decision curve analysis was conducted to determine the clinical usefulness of the nomogram by quantifying its net benefits at different threshold probabilities in the validation cohort.

Using the FRS, we evaluated the risk of incident CVD events over the subsequent 10 years for all subjects based on the factors of age, sex, current smoking status, blood pressure, TC levels and HDL-c levels. Finally, we compared our clinical prediction model based on LR analysis with the FRS in terms of its sensitivity, specificity and AUC. A receiver operating characteristic curve was drawn to evaluate the clinical value of the prediction model in RA patients. All data were analyzed with SPSS 23.0 software and R software.

## Supplementary Material

Supplementary Figures

Supplementary Tables
