# Supplementary material for: Development and validation of a nomogram to predict coronary heart disease in patients with rheumatoid arthritis in northern China
Source: Aging (Albany NY). 2020 Feb 29;12(4):3190–204. doi: 10.18632/aging.102823 (PMC7066926; doi:10.18632/aging.102823)
Supplement: Supplementary Figures [file aging-12-102823-s002..pdf]

SUPPLEMENTARY FIGURES

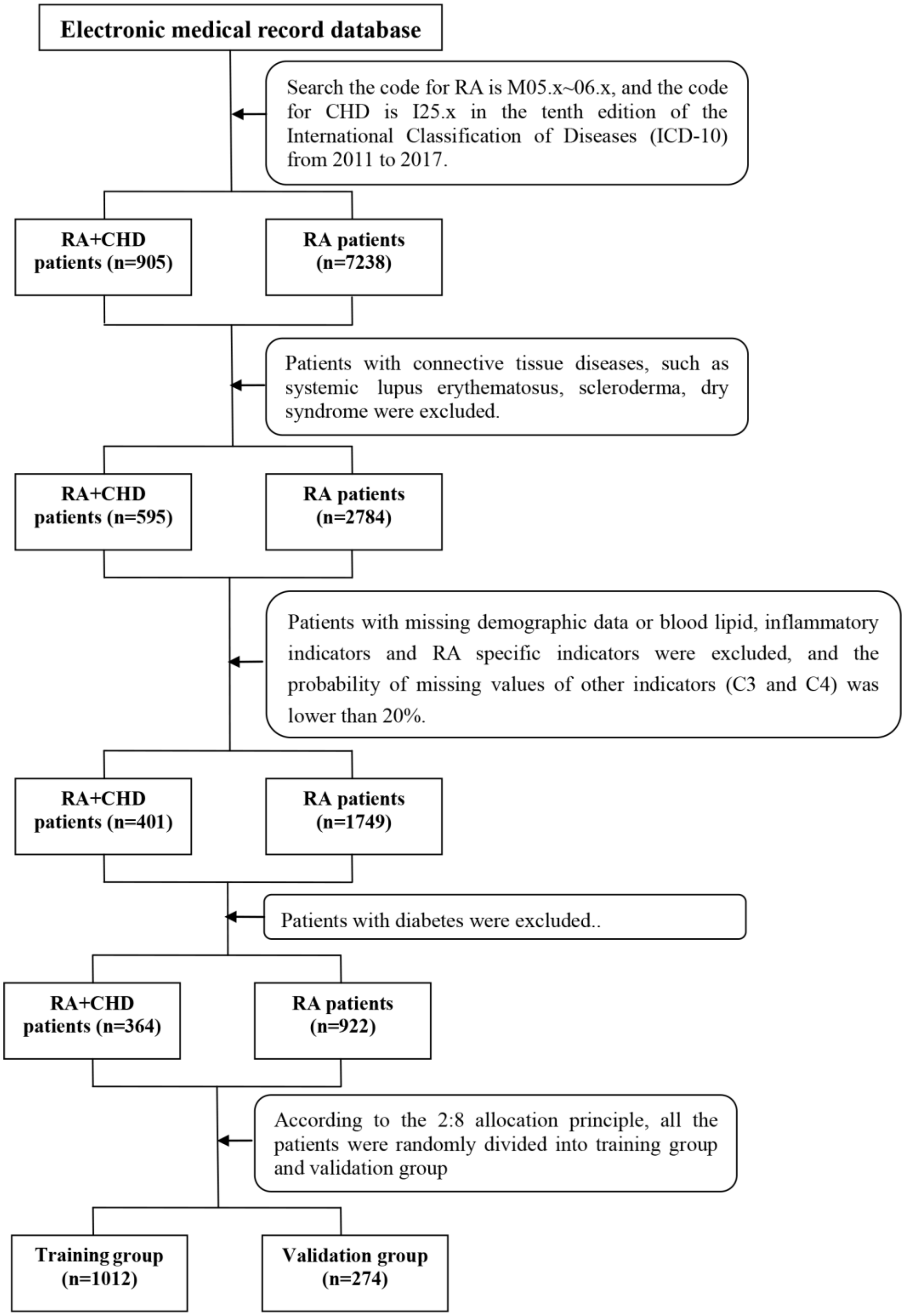

Supplementary Figure 1. Flow chart showing the selection process of study participants. Abbreviation: RA, rheumatoid arthritis; CHD, coronary heart disease.

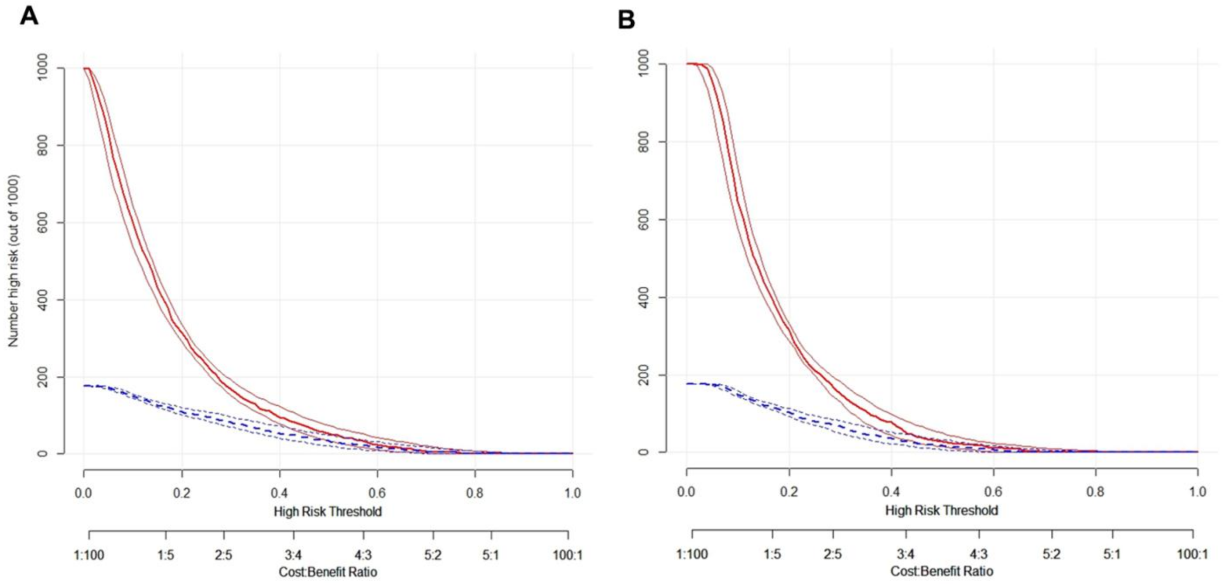

Supplementary Figure 2. The clinical impact curves of the complex model (**A**) and the complex model (**B**) predicting risk stratification of 1,000 people and showing the loss-benefit ratio. The red line, number high risk: under each threshold probability, the number of people classified by simple or complex model as positive (high risk). The blue line, number high risk with outcome: the number of people with true positive under each threshold probability. The y-axis represents the net benefit, the x-axis represents the high risk (out of 1000) of CHD in RA patients. In this example, the dotted vertical line illustrates a tentative cut-point (18% risk of CHD), at which there are 400 of 1000 of the men (40%) would be filtered by simple model, with about 160 of these (40%) being true CHD cases, and 380 of 1000 of the men (38%) would be filtered by complex model, with about 160 of these (42%) being true CHD cases.
