# Supplementary material for: Development and validation of a nomogram to predict coronary heart disease in patients with rheumatoid arthritis in northern China
Source: Aging (Albany NY). 2020 Feb 29;12(4):3190–204. doi: 10.18632/aging.102823 (PMC7066926; doi:10.18632/aging.102823)
Supplement: Supplementary Tables [file aging-12-102823-s001..pdf]

SUPPLEMENTARY TABLES

Supplementary Table 1. Model evaluation of six machine learning models.

	Training group						Validation group					
	auc	accuracy	f1	recall	precision	ber	auc	accuracy	f1	recall	precision	ber
**Simple model**												
GBDT	0.80	0.76	0.60	0.61	0.65	0.39	0.74	0.76	0.56	0.58	0.58	0.42
KNN	0.92	0.91	0.86	0.86	0.90	0.14	0.74	0.76	0.56	0.56	0.68	0.44
LR	0.79	0.71	0.68	0.70	0.68	0.30	0.76	0.69	0.65	0.69	0.65	0.31
RF	0.91	0.81	0.73	0.72	0.83	0.28	0.75	0.76	0.63	0.63	0.72	0.37
XGB	0.81	0.77	0.67	0.66	0.74	0.34	0.74	0.77	0.65	0.63	0.70	0.37
SVM	0.78	0.72	0.69	0.71	0.68	0.29	0.75	0.69	0.65	0.70	0.65	0.30
**Complex model**												
GBDT	0.97	0.91	0.90	0.91	0.89	0.09	0.77	0.73	0.65	0.67	0.66	0.33
KNN	1.00	1.00	1.00	1.00	1.00	0.00	0.78	0.75	0.53	0.54	0.66	0.46
LR	0.79	0.76	0.69	0.69	0.72	0.31	0.79	0.76	0.68	0.68	0.70	0.32
RF	0.96	0.89	0.87	0.88	0.86	0.12	0.76	0.73	0.65	0.66	0.65	0.34
XGB	0.92	0.86	0.82	0.80	0.86	0.20	0.76	0.75	0.63	0.62	0.65	0.38
SVM	0.81	0.75	0.72	0.74	0.71	0.26	0.79	0.72	0.68	0.72	0.67	0.28

Abbreviation: GBDT:gradient boosting decision tree; KNN: k-nearest-neighbors; LR: logistic regression; RF: random forest; XGB: xgradient-boosting; SVM: support vector machine.

Supplementary Table 2. Risk factors for RA patients developing to CHD in the training group.

	Simple model		Complex model (adjusted age and sex)	
	OR(95% CI)	*P*	OR(95% CI)	*P*
Age (year)	-	-	1.05(1.04,1.07)	0.000
Sex (male, %)	-	-	0.88(0.6,1.28)	0.503
Hypertension(%)	3.06(2.26,4.15)	0.000	2.95(2.15,4.05)	0.000
Anti-CCP-positive(%)	1.32(0.95,1.82)	0.096	1.41(1.00,1.97)	0.047
RF-positive(%)	1.48(1.04,2.11)	0.031	1.36(0.94,1.97)	0.099
ESR(mm/h)	1.01(1.00,1.01)	0.001	1.01(1.00,1.01)	0.009
CRP(mg/L)	1(0.99,1.00)	0.958	1.00(1.18,1.80)	0.880
LDL(mmol/L)	1.85(1.25,2.72)	0.002	1.95(1.31,2.91)	0.001
TC(mmol/L)	0.79(0.54,1.14)	0.209	0.77(0.53,1.12)	0.166
HDL(mmol/L)	2.17(1.26,3.76)	0.006	2.04(1.16,3.61)	0.013
TG(mmol/L)	1.41(1.14,1.74)	0.002	1.47(1.19,1.81)	0.000
Constant	0.017	0.000	0.001	0.000

Abbreviation: LDL, low-density lipoprotein cholesterol; TC, total cholesterol; TG, triglycerides; HDL, high-density lipoprotein cholesterol; RF-positive, positive rheumatoid factor; CRP, C-reactive protein; Anti-CCP-positive, positive anti-cyclic citrullinated peptide antibody; ESR, erythrocyte sedimentation rate.

Supplementary Table 3. Sensitivity and specificity of FRS and the prediction model in detecting CHD.

Variables	AUC	*P*	95%CI	Cut-off %	Specificity	Sensitivity
FRS	0.66	0.000	0.62-0.70	3.5% [a]	52.4%	71.8%
PRE1	0.73	0.000	0.69-0.76	23.7%	59.2%	73.8%
PRE2	0.77	0.000	0.74-0.80	32.8%	77.2%	63.9%

AUC, area under the curve; CI, confidence interval; [a] 10-year cardiovascular disease risk
